# The Added Benefit of Combining Laser Doppler Imaging With Clinical Evaluation in Determining the Need for Excision of Indeterminate-Depth Burn Wounds

**DOI:** 10.7759/cureus.8774

**Published:** 2020-06-22

**Authors:** Mohammed Asif, An Guo Michael Chin, Tomer Lagziel, Kevin M Klifto, Ashley D Modica, Eliana Duraes, Julie Caffrey, Charles S Hultman

**Affiliations:** 1 Plastic Surgery, Johns Hopkins University School of Medicine, Baltimore, USA; 2 General Surgery, St. John's Episcopal Hospital, Far Rockaway, USA; 3 Medicine, Tel-Aviv University, Sackler School of Medicine, Tel-Aviv, ISR; 4 Plastic Surgery, University of South Florida (USF) Health, Tampa, USA

**Keywords:** laser doppler imaging (ldi), indeterminate depth burn wounds, digital health communications, digital images, burn surgery, second degree burn, burn, wound care

## Abstract

Background

Managing indeterminate-depth burn wounds remains challenging. Laser Doppler Imaging (LDI) has been validated for burn wound depth and can influence the clinical assessment. Our study investigated the value of LDI as an adjunct in determining the need for excision.

Methods

Seventy American Burn Association (ABA)-verified burn centers were surveyed. A controlled pre-test assessment without LDI and post-test assessment with LDI of 100 indeterminate-depth burn wounds was conducted to evaluate the influence on the clinical judgment among different health professionals. Relative risk, analysis of variance (ANOVA), paired t-test, and intention-to-treat were used for analysis. A p-value $\leq$ 0.05 was considered significant.

Results

Among 32 burn centers, three confirmed using LDI. Six thousand grader-image interactions were analyzed. There was a significant difference in the predictive accuracy for pre-LDI and post-LDI assessments when all graders were considered (51.9% ± 7.0 vs. 72.9% ± 7.9; p < 0.0001). Post-LDI assessment added 20.9% more accuracy than the pre-LDI assessment. The post-LDI assessment was 1.4 times more likely to correctly predict the need for excision and skin-grafting than the pre-LDI assessment. All groups had an improved performance post-LDI: Group 1 (physicians), 51.9 ± 7.5 versus 76.4±5; Group 2 (nurses), 52.1 ± 6.1 versus 72.7±7.7; and Group 3 (others), 51.7 ± 9.2 versus 68.6 ± 10.1. No statistical difference was observed between groups (p = 0.92).

Conclusion

LDI makes the clinical examination of indeterminate-depth burn wounds more accurate. For every five LDI evaluations performed, one assessor changed their treatment plan as a result of this imaging technique. LDI is cost-effective and increases the accuracy of determining the severity of indeterminate-depth burn wounds.

## Introduction

Accurate visual assessment of burns is an integral step in the treatment plan process for indeterminate-depth burn injuries. Yet, visual assessment of burns has remained relatively unchanged since the 16th century [1]. With the recent upsurge of telemedicine around the world, many healthcare providers are accepting the use of digital health communications in their medical practices [2]. Digital health communications are becoming especially popular in burn centers because of their improved convenience, accuracy, efficiency, productivity, and clinical decision-making [3]. Telemedicine is constantly explored for placements and improvements in healthcare settings, but its integration into practice can often be difficult to implement [4].

Traditionally, verbal or written burn wound descriptions were the sole source of communication between healthcare professionals with limitations of subjectivity, which hindered accuracy. Yet, when digital images were introduced, the accuracy of burn assessments improved dramatically [5]. It is more efficient to make an assessment from a digital image than from a provided wound description from other healthcare professionals, especially non-burn specialists [2].

However, digital images are not perfect in assessing burn wounds since they lack important information such as burn depth. Like a naked eye, a digital image has its limitation of a two-dimensional view of a burn wound surface and cannot provide a visual of the deeper layers of the skin [6]. Two-dimensional (2D) digital photographs are limited because they have been shown to increase validity in size assessment, but not depth assessment [7]. Even though a 2D view of a burn wound can provide good information, having tools that provide additional dimensions of assessment would further improve clinical burn wound outcomes.

There have been numerous technological developments to assist in managing burn wounds, including Laser Doppler Imaging (LDI), which is a helium-neon laser that objectively measures the blood flow and/or healing potential for indeterminate-depth burn wounds. LDI is a non-invasive mechanical extension of the clinical physical exam for burn wounds because it objectively identifies burns that have good or poor wound healing potential to help decide whether or not to proceed with surgical excision and grafting.

Even though studies indicate that visual evaluation of digital images is accurate enough for clinical decision-making, there are not sufficient studies looking into the accuracy of assessing digital images of indeterminate-depth burn wounds [8]. The reliability of making a preliminary indeterminate-depth burn wound assessment based on digital images should be further explored due to its two-dimensional limitations.

We investigated how additional information from LDI can influence the clinical assessment of indeterminate-depth burn wound digital images. The aim of this study was to investigate the accuracy of burn wound photographs and the value of LDI as an adjunct to clinical evaluation in determining the need for excision of 100 indeterminate-depth burn wounds among different healthcare providers.

## Materials and methods

We conducted a controlled pre-test/post-test assessment at a burn center to evaluate the influence of LDI on the clinical judgment of physicians (Group 1), nurses and physician assistants (Group 2), and other health professionals (Group 3). 

**Figure 1:**
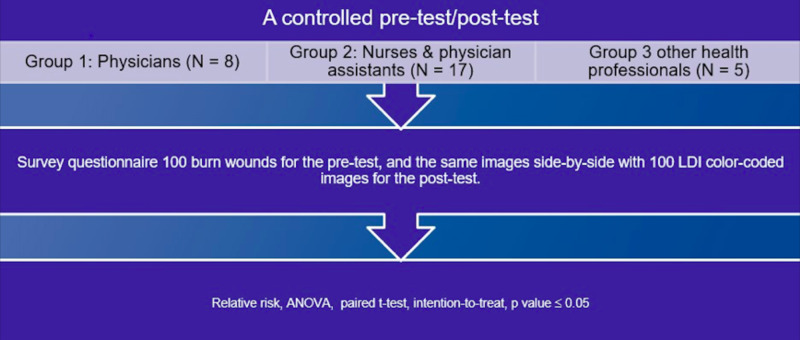
A controlled pre-test/post-test assessment algorithm ANOVA, analysis of variance; LDI, Laser Doppler Imaging

The study involved a retrospective review of 100 patients over three years and inclusion criteria included patients aged 18 years or older with indeterminate-depth burns who received an LDI scan. The controlled assessment consisted of regular digital images of 100 burn wounds for the pre-test, and the same images side-by-side with 100 LDI color-coded images for the post-test. LDI was performed, on average, 2.81 days after the injury was sustained. For burn wound management, the historical decision of two senior burn surgeons was considered standard of care. The determination of surgical versus non-surgical intervention was based on these surgeons’ expert opinion after clinical and technological evaluation. A burn surgeon’s expert opinion is a culmination of academic knowledge and professional, clinical experience. Relative risk, analysis of variance (ANOVA), paired t-test, and intention-to-treat were used for analysis. A p-value $\leq$ 0.05 was considered significant (Figure 1).

**Figure 2:**
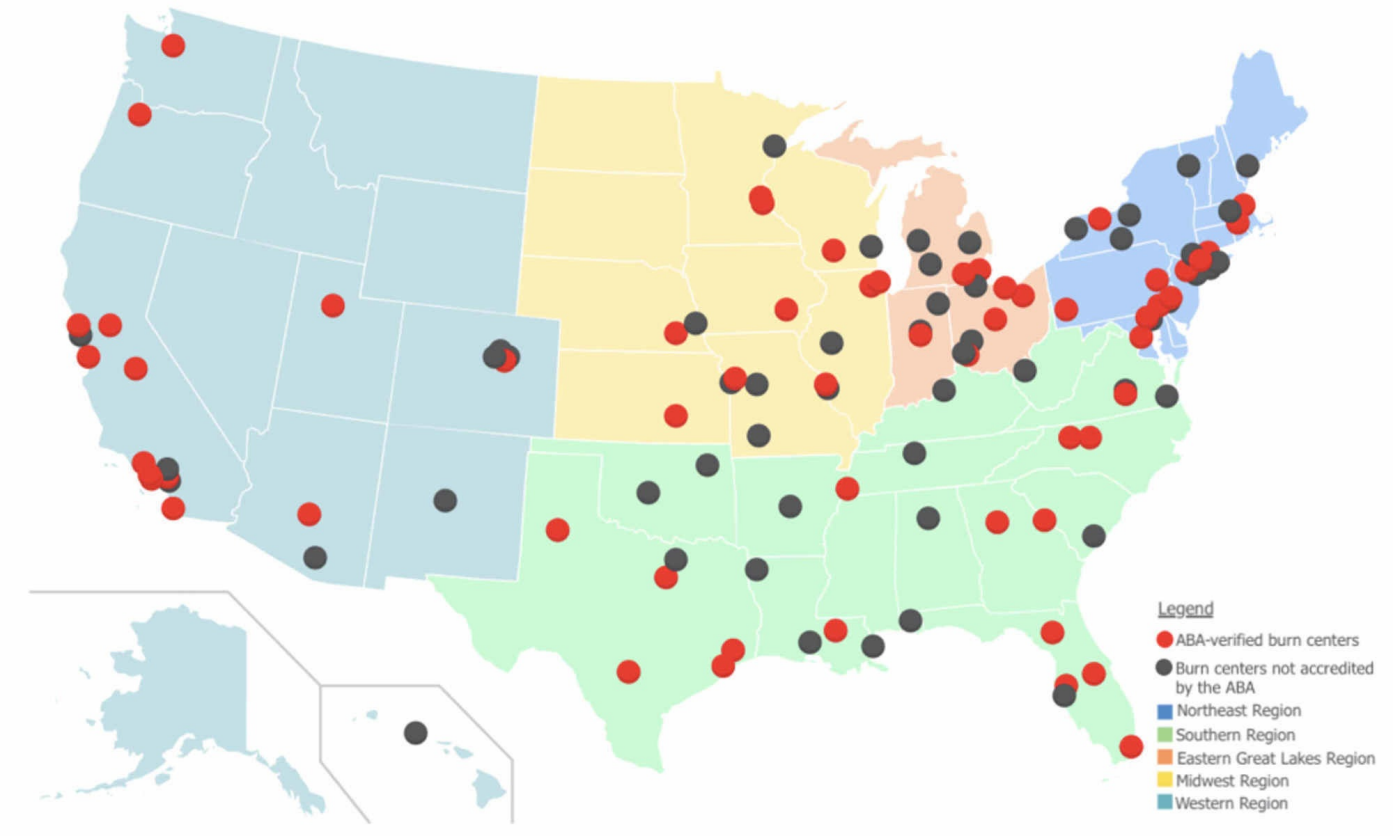
Map of ABA-verified burn centers across the United States Adapted from the American Burn Association [9]

We also carried out a survey of 70 American Burn Association (ABA)-verified burn centers on the utilization of LDI methods (Figure 2). The survey was a basic questionnaire of ABA burn centers to understand if they implement LDI technology in their analysis and treatment plan of indeterminate-depth burn wounds.

## Results

**Figure 3:**
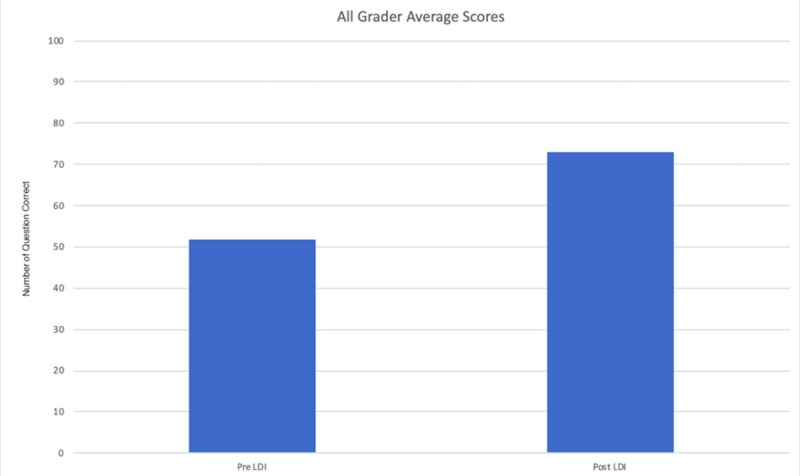
Pre-/post-LDI assessments among different healthcare providers LDI, Laser Doppler Imaging

**Figure 4:**
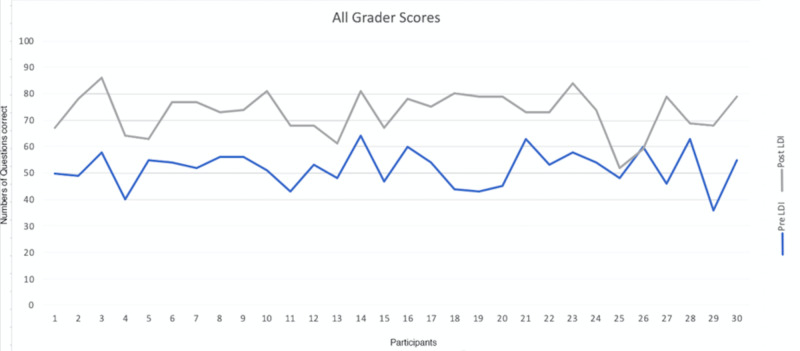
Post-LDI assessment improved among different healthcare providers LDI, Laser Doppler Imaging

**Figure 5:**
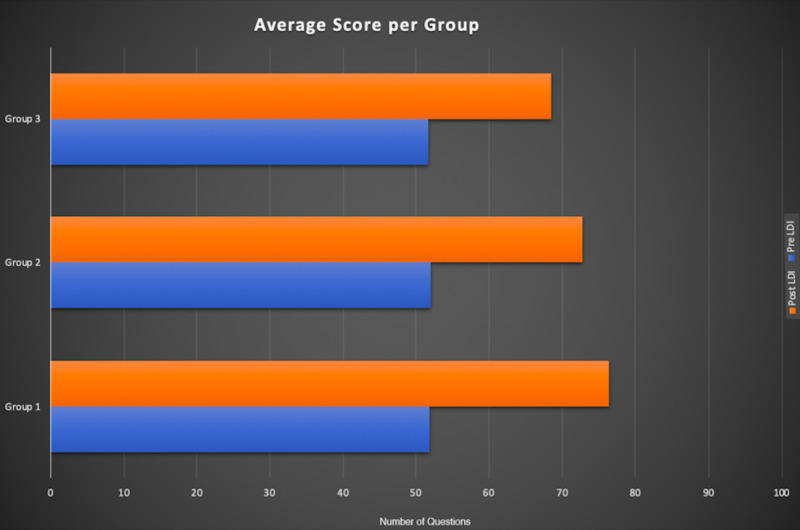
Breakdown of pre-/post-LDI assessments among different healthcare providers LDI, Laser Doppler Imaging

A group of 30 professionals analyzed 100 digital images with and without LDI resulting in a total of 6,000 grader-image interactions were analyzed. There was a significant difference in the predictive accuracy for pre-LDI and post-LDI assessments when all graders were considered (51.9% ±7.0 vs. 72.9% ±7.9; p < 0.0001) (Figure 3). On average, the post-LDI assessment was 20.9% more accurate than the pre-LDI assessment (95% CI [17.388, 24.478]). The post-LDI assessment was 1.4 times (RR = 1.4, CI [1.12-1.75]) more likely to correctly predict the need for excision and skin grafting than the pre-LDI assessment alone. All professional groups had an improved performance post-LDI: Group 1, 51.9 ± 7.5 versus 76.4 ± 5; Group 2, 52.1 ± 6.1 versus 72.7 ± 7.7; and Group 3, 51.7 ± 9.2 versus 68.6 ± 10.1; no statistical difference was observed between groups (p = 0.92) (Figures 4, 5).

Survey results demonstrated that of the 32 ABA-verified burn centers who responded, only five confirmed using supplemental digital technology, three of which were LDI. The other modalities were infrared spectroscopy and thermography.

## Discussion

The field of burn surgery has evolved slowly in the past century when comparing to other surgical specialties as evident by our telephonic survey. There were only five ABA-verified burn centers that used supplemental technology to adjunct their clinical assessment of the indeterminate-depth burn wounds. Our study demonstrated that the use of high-resolution JPEG images yielded accurate results in only 51.9% of the cases and after evaluating the same images with LDI assessment, the predictive accuracy improved 20.9% among different health providers. Notably, for every five LDI evaluations, one assessor changed their treatment plan in response to LDI, resulting in less surgical interventions, decreased length of stay, less opioid prescriptions, and lower overall morbidity. In 2018, a study published by King Edward Medical University and Mayo Hospital in Pakistan comparing the accuracy of LDI and clinical assessment in determining wound depth showed 90.21% diagnostic accuracy, 92.75% sensitivity, 82% specificity, 94% positive predictive value, and 79% negative predictive value. The results for clinical assessment were 81.52%, 81%, 82%, 93%, and 59%, respectively [8]. Burn surgery and wound management involve a multidisciplinary treatment team. This highlights the importance of our methodology involving review by non-physicians and physicians. The standard non-LDI approach involves a more aggressive and less precise surgery. The change resulted from LDI intervention involved either a non-invasive treatment approach or a more targeted surgery. The more effective healing allowed for a shorter hospital stay.

The majority of developments in the field have occurred relatively recently, due to an increase in burn research, better understanding of wound physiology, and new surgical and critical care techniques [10]. Many technologies are emerging for burn care with LDI at the forefront of clinical burn depth assessment, having the ability to evaluate microvascular dermal perfusion [11]. To highlight its effectiveness, LDI has even been validated in a multi-center study, examining over 400 wounds and yielding 96% technical accuracy in predicting healing time [12]. Other studies that have compared LDI and other imaging technologies have demonstrated significantly more accurate results with LDI [13].

The primary competitor to LDI in the field of digital imaging assessment is thermal imaging technology. This not-so-modern technology dates back to the 1960s and maps skin temperatures using infrared scanning techniques [14]. Today, LDI and thermal imaging are the two primary adjuncts to assist in clinical assessments of indeterminate burns due to their complex nature. However, comparative studies have shown that LDI is a far more accurate assessment tool [15]. Yet, due to significantly lower costs, thermal imaging is used more than LDI, both clinically and in research [13]. Thermal imaging techniques are also preferred due to their ease-of-use, instantaneous image production, and device portability [16]. The primary limitation arises from the fact that the image produced by thermal imaging devices is greatly influenced by environmental factors like wound evaporation and humidity [17]. In addition, new improvements in LDI have made devices easier to move, images more accurate, and faster scanning times [18]. LDI was developed for the objective measurement of perfusion in indeterminate-depth burns to better assess the need for surgery. Despite these clear advantages, the integration of LDI into US burn centers in not nearly as widespread as those in the UK, primarily due to cost [19]. This is further supported by our survey, which indicates that not many burn centers utilize LDI nor do they plan to. A thorough financial assessment of LDI devices was performed in the Netherlands and it showed that with an initial device investment of €57,590 ($63,570), an average of €875 ($1080) per patient could potentially be saved due to decreased hospital stay [20]. We contacted Moor Instruments (Axminster, UK), the manufacturer and distributor of LDI imaging systems in the UK, and an internal representative reported that a single LDI device could be purchased for as little as $60,000, which could generate significant hospital savings. In addition to reducing hospital costs, the use of LDI in indeterminate-depth burn assessment should improve the patient experience by decreasing unnecessary surgical operations, reduced length of hospital stay, and fewer follow-up hospital visits [21,22]. At Johns Hopkins Bayview Medical Center, the estimated average direct cost of an inpatient procedure is approximately $14,000 [23]. While we did not collect specific costs for each patient in our study, estimated expenses can help conclude that using LDI to avoid extended hospitalization and unnecessary procedures will help the hospital and the patient avoid excessive expenses.

In the United States, burn surgeons continue to use traditional approaches in managing their patients, whether it is local wound care or excision and grafting [24]. In our survey of 70 ABA-verified burns centers, only three out of 32 (9.4%) responded confirming using LDI to evaluate burn wounds, which is slightly higher than the 6% reported in a survey from 2014 [19]. Even with this slight increase in usage, the use of LDI is significantly lower than in European burn centers (32%) [7]. Widespread European LDI integration could be due, in part, to the official statement by the National Institute for Clinical Excellence (NICE) in the UK that LDI devices provide a clear clinical benefit in guiding clinical decisions for indeterminate-depth burns and predicting healing outcomes [25]. 

Medical technology is rapidly advancing in recent years with constantly emerging tools targeted for enhancing patient care. US burn centers have not been quick to adapt to new technologies even in light of their added benefits, which ultimately hinders patient care. The need for novel adjunct interventions is crucial, especially when evaluating indeterminate burns. Due to their indeterminate nature, they are significantly more complex, requiring more time for clinical assessment, which leads to a delay-to-operation, resulting in a longer hospital stay [26-28]. When preceded by LDI, surgical intervention for indeterminate-depth burn injuries allows for a more accurate course of treatment and ultimately a more positive patient experience [29,30]. From our experiences using LDI in the clinical setting, we can confidently confirm that the increased clinical efficiency that results from adjunct LDI-use is beneficial to both the hospital and the patient.

Our study carries a few limitations including the assumption that the two senior burn surgeons made correct and accurate decisions in managing their burn patients; their professional decision is still subjective relative to their experience. While the expert opinion of the senior burn surgeons was considered standard of care, 22 non-surgeons evaluated images because it is crucial to demonstrate that LDI-use is effective even when used by non-physician medical personnel. In most burn centers, the patient is treated by a multidisciplinary team and the technology at hand should be suited for that staff. The effect of LDI utilization on cost-effectiveness, patient-care, and safety has not been thoroughly examined but we hope that this study will prompt further examination. Our study is a single-center study but we plan to expand this type of research to additional burn centers across the United States. The plan for the future is a multi-institutional study and other LDI institutions have been contacted for future prospective studies. We estimate that once these outcomes are further assessed on a nation-wide approach, the positive outcomes will pave the way for advancing the field of burn surgery in the United States. However, we are aware that every medical institution manages its financial budget independently so we cannot infer what calculations are considered in evaluating large expenses. Unfortunately, given the complex nature of burn wounds and the patient population involved, no proper follow-up assessing healing outcomes was performed. Finally, a technical limitation exists given that when analyzing digital photographs, there is no imaging quality standard in practice. This means that some of the images analyzed were captured by different devices at different quality levels.

## Conclusions

Color-coded LDI images aid in the clinical judgment of digital photographs and make the clinical examination of indeterminate-depth burn wounds more accurate. As observed, through the utilization of LDI technology as a supplemental analysis tool, the operative plan was changed from surgical to non-surgical intervention. For every five LDI evaluations performed, one assessor changed their treatment plan as a result of the LDI. This means that approximately 80% of surgeries proceeded as originally planned. Other studies have demonstrated that LDI is more cost-effective and is more accurate than competing imaging techniques like thermal imaging. Our study showed that digital photos alone are unreliable in predicting burn severity and the need for excision. LDI, as a supplemental tool, is cost-effective because, based on estimated patient charges and hospital expenses that can be avoided, LDI devices will pay for themselves very quickly and will increase the accuracy of determining the severity of burn wounds while significantly improving patient outcomes.
